# Preparing for the EU HTA Regulation: Insights from the Dutch Perspective

**DOI:** 10.3390/jmahp13030035

**Published:** 2025-07-24

**Authors:** Anne Willemsen, Maureen Rutten-van Mölken, Riam al Dulaimi, Hedi Schelleman, Wim Goettsch, Lonneke Timmers

**Affiliations:** 1 Zorginstituut Nederland, 1112 Diemen, ZA, The Netherlands; 2 Utrecht WHO Collaborating Centre for Pharmaceutical Policy and Regulation, Division of Pharmacoepidemiology and Clinical Pharmacology, Utrecht University, 3584 Utrecht, CG, The Netherlands; 3 Erasmus School of Health Policy and Management, Erasmus University Rotterdam, 3062 Rotterdam, PA, The Netherlands; m.rutten@eshpm.eur.nl

**Keywords:** EU HTA regulation, EU HTA process, HTA body, HTA procedure

## Abstract

The European Health Technology Assessment (HTA) regulation (HTAR) came into effect in January 2025 and impacts the HTA process in all European Member States. Member States must give due consideration to the joint clinical assessment (JCA) report. This may require adaptations at the national level. This paper describes the anticipated changes to the Dutch national HTA process and how the Dutch National Health Care Institute (Zorginstituut Nederland, ZIN) prepared for this, because sharing experience between Member States can be of general interest for future expansion of the EU HTAR. ZIN’s implementation activities were facilitated by a project-governance structure and by a continuous gap analysis of the current national assessment and appraisal process of medicinal products, resulting in a concrete action plan. The implementation of the HTAR has two major implications for ZIN’s HTA process, namely that the scoping phase starts much earlier and that the JCA report is the starting point for the national assessment. Gaps, challenges and issues were identified in the categories: information and knowledge, IT and template, communication and stakeholder engagement, capacity and resources, and financial aspects. Based on a thorough and well-defined implementation plan, ZIN is ready to implement the HTAR in national HTA processes and to take on (co-)assessor roles for JCA of medicinal products in 2025.

## 1. Introduction

After many years of preparatory work, the European Health Technology Assessment (HTA) regulation (EU) 2021/2282 (HTAR) was adopted in 2021 and came into effect in January 2025 [1], defining a legal framework for European HTA. Key components of the HTAR are Joint Scientific Consultations (JSCs) and Joint Clinical Assessments (JCAs), for medicinal products, medical devices (MDs) and in-vitro diagnostic medical devices (IVDs) that fall within the scope of the HTAR [1].

The application of the HTAR will impact the HTA processes in all EU Member States (MSs) as MSs must give due consideration to JCA reports in their national HTA work. The HTAR defines obligations for the MS regarding the JCA, for example, with regard to the data they can and cannot request from the Health Technology Developer (HTD) on the national level. Please refer to Appendix A for details on these obligations and further explanation.

The JCA process of medicinal products starts in parallel with the European Medicines Agency (EMA) process for granting marketing authorisation, leading to a timely availability of a JCA report for the national appraisal phase. The JCA focuses on the assessment of relative effectiveness and relative safety and results in a JCA report that gives a factual and objective description of this data against alternative treatment options—both pharmaceutical and non-pharmaceutical—as requested by the EU MSs. Contrary to the European process of market authorisation, reimbursement decisions will remain to be a matter of national responsibility, also under the HTAR [1]. With this JCA report as a common starting point, all MSs still have the freedom to draw their own conclusion on the therapeutic benefit of an intervention, taking into account the national context. Furthermore, they may have to perform a country-specific appraisal of the non-clinical evidence, such as cost-effectiveness, budget impact, and ethical, legal, social and organisational consequences before making the national reimbursement decision.

The Dutch National Health Care Institute (ZIN), i.e., the Dutch HTA body, which has the overall responsibility for governing the publicly reimbursed health insurance package in the Netherlands, has a longstanding history when it comes to European collaboration in the field of HTA. In 2009, ZIN was one of the founding partners of the European Network for HTA (EUnetHTA). It was the project leader of the work packages on Relative Effectiveness Assessments in Joint Action 1 and 2, has taken on the overall coordination of Joint Action 3 in 2016 [2,3,4,5], and has been acting as consortium lead in EUnetHTA 21 [6]. This active role continues with ZIN serving as the designated Dutch member organisation within the HTA Coordination Group (HTACG) and an active participant in all of its subgroups. ZIN co-chairs the subgroup on JCA.

Since 2021, HTA bodies of the MSs have been preparing for participation in the HTACG and the different subgroups, particularly the JSC and JCA subgroups and procedures. How the MSs will implement the HTAR may differ between MSs, as it depends on their current HTA processes and guidelines. Yet, in informal exchanges ZIN had with other HTA bodies, it became clear that many have similar questions around implementing the HTAR and optimising the national HTA timelines to give due consideration to the JCA report. We believe that it is of general interest to describe how ZIN has prepared for the implementation of the HTAR in the Dutch setting and taking up a role as (co-)assessor for a JCA, for MSs dealing with similar challenges now and potentially in the future when the HTAR is expanded to include orphan designation products in 2028 and all centrally authorised medicinal products in 2030. In this paper, we briefly describe the current national HTA process and the expected impact of the HTAR on this status quo. Furthermore, we explain how the governance of the national implementation was organised and report the results of a gap analysis. Finally, we describe the actions taken to address the gaps and move to a system in which the results of the HTAR are efficiently implemented and used to inform national reimbursement recommendations for medicines and medical devices.

## 2. Materials and Methods

ZIN applied structured change management to develop a framework for understanding, managing and facilitating organisational change during the period 2022 to 2024. This framework started with developing a clear vision on the desired outcome. With this end goal in mind, reverse engineering was applied to identify the gaps and the necessary steps and initiatives to address the gaps. For each of these steps, assumptions about what is required for change to occur were made and the causal mechanism through which each initiative would logically contribute to the desired outcome was specified. Theories of change management stress the importance of participation, communication, and buy-in from those affected [7]. ZIN therefore adopted a governance structure that involved the respective staff members in co-designing the change process, including a process of continuous learning in which progress was monitored, feedback was provided and corrections to the course of action were made when necessary. External stakeholder engagement was also part of this process, both to inform the stakeholders about anticipated changes and to collect input on additional gaps, issues or challenges relevant for them.

### 2.1. Desired Outcome

ZIN’s preparatory activities were undertaken in light of the implementation of the HTAR in the national process and ZIN’s ambition to take on an active role as (co-)assessor in the JCA process, which followed from ZIN’s active participation throughout the various EUnetHTA iterations. Hence, the desired outcome was a national HTA process that enables efficient contributions to the JCA process and efficient use of the JCA reports in the national HTA process. ZIN focused on medicinal products because when the preparation started, it was unclear when JCAs for MDs and IVDs would start, and these MDs are not subject to mandatory assessments by ZIN.

### 2.2. Governance of ZIN’s Change Process

To lead the preparatory activities for the HTAR implementation, ZIN has set-up a horizontal project governance in 2022. The idea behind this governance structure was to co-design the change process, involving all relevant stakeholders within ZIN and ensuring a broad communication of the HTAR and the anticipated changes within ZIN. In addition, it prepared the representatives of ZIN for their role in the HTACG and subgroups, and aligned their contributions. The governance structure involved a project lead and a project team that was divided into a sub-team focusing on changes in the HTA process and a sub-team focusing on changes in HTA methodology. The project team was supervised by a Steering Committee and advised by an advisory group. For each of these entities, the number and type of staff members involved, their tasks and the frequency of meeting was defined. In addition, internal meetings with ZIN representatives in the HTACG, the four subgroups and the HTA Comitology Committee were held. This entire structure enabled organisation-wide continuous monitoring and feedback.

### 2.3. Gap Analysis

One of the main activities of the project team was to conduct an iterative and continuous gap analysis of the current national process of assessment and appraisal of medicinal products in order to identify changes that might be needed to accommodate the HTAR with the focus on JCAs. ZIN adopted a practical approach to conduct a gap analysis. A first workshop conducted with the project team led to an initial classification of the topics to be addressed during the gap analysis. The Steering Committee also played a crucial role in the gap analysis, not only because of their decision-making ability but also because they identified additional potential gaps, challenges or issues as well as appropriate actions required. Lastly, the exchanges with external stakeholders led to the identification of further questions to be explored.

The gap analysis focused both on the process of EU HTA as well as the methods used to conduct EU HTA. In this gap analysis, ZIN examined which gaps, challenges or issues could arise when using the JCA report in the national setting. To start this gap analysis, comparisons were made between the EU HTA process and methods (since the gap analysis started in 2022, the early version was based on procedures and guidances as developed under EUnetHTA 21 because the guidances were not officially adopted under the HTACG yet) and those at the national level.

As the project started before any of the Implementing Regulations were available and before any guidance was adopted by the HTACG, the gap analysis was conducted and monitored on a continuous basis to keep up with elaborations and capture relevant details at later stages.

For each identified gap, issue or challenge, actions to address them were developed and agreed upon, with timelines established for implementation. This action plan was discussed within the entire project team by exploring how the actions were expected to contribute to solving the gaps, and finally this action plan was approved by a Steering Committee at ZIN.

### 2.4. External Stakeholder Engagement

A key activity in the preparatory activities from ZIN was to prepare external national stakeholders, such as the association of innovative medicines developers, the healthcare insurers, patient organisations and clinical societies for the new situation and the role they were anticipated to have or how it will impact them. These interactions occasionally also led to the identification of additional gaps, issues or challenges for our project.

Part of ZIN’s activities was to have informal exchanges with several other HTA bodies across Europe to understand how they were preparing and what their key questions where. Within the Beneluxa collaboration, the preparations for HTAR were discussed within the domain taskforce HTA. While the gaps identified will likely differ between MSs because of the difference in the HTA processes in different MSs [8], many of the more overarching questions regarding the implementation of the HTAR on the national level were expected to be similar.

## 3. Results

### 3.1. Short Description of the National Process for Medicinal Products

In the Netherlands, ZIN is responsible for governing the basic health insurance package of the Dutch mandatory health insurance. It conducts assessments and provides frameworks and guidelines. Standard tasks are HTA and advice on reimbursement for all out-patient medicines and, via a risk-based approach, for hospital medicines which meet certain criteria (in 2024, the criteria for entering the lock procedure were that the total costs for one new indication, or multiple new indications combined, are expected to exceed EUR 20 million per year. If total costs for one new indication are expected to be≥ EUR 50.000 per patient per year, and total costs are expected to exceed EUR 10 million per year, the indication will also be placed in the lock procedure). If the costs and budget impact of hospital medicines are high, like for many oncology medicines and advanced therapeutic medicinal products (ATMPs), these drugs enter the ‘lock procedure’ to be able to manage the introduction into the basic health insurance package [9]. Medicines placed in the lock procedure need to be assessed by ZIN and their price may be negotiated later which is usually done by the Dutch Ministry of Health and the HTD. All hospital medicines that do not meet the lock criteria are handled by the joint Dutch health care insurers. For this, they collaborate in the Committee Assessment of Add-on Medication (CieBAG) [10], which decides whether the medicine will be included in the basic health insurance package. The position of the Dutch health insurers is different from ZIN, and their process of assessment is less extensive.

The national HTA report prepared by ZIN is based on a submission by the HTD and typically includes a pharmaco-therapeutic assessment report, often accompanied by a budget impact analyses, and may also include a pharmaco-economic report. In case a full HTA is performed and the complete process is carried out, the following steps are taken. ZIN drafts three separate reports: a pharmaco-therapeutic report, a budget-impact analysis and a pharmaco-economic report. These draft reports are discussed in the first meeting of the Scientific Advisory Board (WAR). The revised drafts are shared with stakeholders for consultation, including the HTD, the physicians’ association, the patients’ association and the association of Dutch healthcare insurers. Following this written consultation, the draft reports may be adjusted. The revised reports are then reviewed a second time by the WAR. Thereafter, the assessment phase is followed by an appraisal phase with a process of deliberation in the Insured Package Advisory Committee (ACP), which considers the scientific evidence as well as ethical, legal, organisational and other considerations. ACP meetings are public, and live consultation of patients and other stakeholders is part of the process. Finally, based on the input of the ACP, ZIN will issue a recommendation for reimbursement to the Minister of Health, who will, often after price negotiations, come to a final decision on reimbursement. That decision is published in the Netherlands Government Gazette.

### 3.2. Expected Impact of HTAR on the National Process

The implementation of the HTAR has two major implications for the national HTA process: 1) the scoping phase will start much earlier and 2) HTA bodies will have a common starting point for their assessment, namely the JCA report.

First, the scoping phase will start much earlier because the JCA will run in parallel with the regulatory assessment conducted by the EMA. Already in the scoping phase of the JCA, ZIN must submit the PICO (Patients/Intervention/Comparator/Outcome, which constitute the research question for the HTA) or PICOs that it expects to be relevant for the Dutch setting. This is a major change from the regular pre-HTAR process, where defining the national PICO(s) usually starts once the HTD applies for reimbursement in the Netherlands. This might be shortly after market authorisation but can also take months or even years [11]. It should be noted that, in the Netherlands, HTDs have the possibility to submit already before market authorisation. This is the so called ‘parallel procedure’ [12]. Although this is encouraged by ZIN as it contributes to fast access, HTDs are often reluctant to participate in this procedure. Whether the aspired goal of faster availability of innovative medicines to patients is achieved largely depends on the global launch strategy of the HTD.

Second, the JCA report is now the common starting point for the national clinical assessment by HTA bodies. This report lists all available scientific evidence and the degree of certainty of this evidence for the PICOs requested by the MSs. The evidence that is relevant for the Dutch setting will be given due consideration in the pharmaco-therapeutic part of the national HTA report, where it will be complemented with a conclusion about the therapeutic value of the medicine in the national context.

The review of the pharmaco-therapeutic report by the WAR is unlikely to change due to the HTAR, apart from the fact that the WAR will increasingly have to deal with national reports based on information from the JCA reports. Hence, the WAR needs to be duly informed about the new way of working.

**Figure 1:**
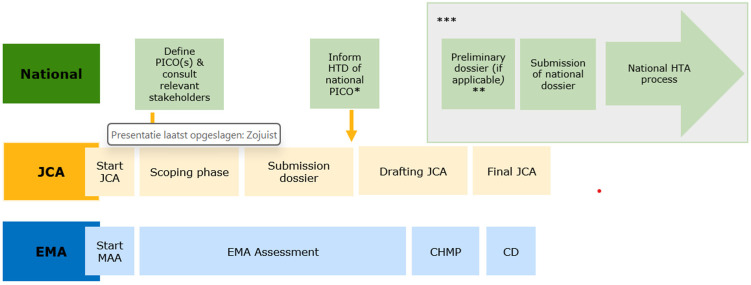
Phases in the HTAR and the national assessment process. Abbreviations: EMA = European Medicines Agency; CHMP = Committee for Human use of Medicinal Products; CD = Commission Decision on Marketing Authorisation; HTD = Health Technology Developer; JCA = Joint Clinical Assessment; MAA = Marketing Authorisation Application; PICO = Patient, Intervention, Comparator(s) and Outcome(s); *: only upon request by the HTD; **: may include a renewed national scoping, this may especially be relevant when the HTD submits much later as the treatment landscape could have changed; ***: it should be noted that this figure presents the earliest timepoints possible for these steps, however, the exact timelines will depend on the national submission by the HTDs.

Figure 1 describes the main steps in the JCA and the national HTA process. Of note, no definitive timelines of the national process are specified, as they depend on when the HTD initiates national submission. In addition, Figure 1 shows the bigger picture of the JCA process and is therefore applicable to both the Standard Marketing Authorisation process and the Accelerated Marketing Authorisation process. The process for so-called Type II variations (i.e., extensions of indications) has not been included in this figure or addressed in the gap analysis because this was also not discussed yet at the European level by the JCA subgroup.

### 3.3. Governance Structure of the Implementation Project at ZIN

To lead the preparatory activities for the HTAR implementation, ZIN set up a horizontal project governance in 2022. The two main departments that played a key role within this project were Healthcare (Zorg) and Research and Development & International Affairs (OWIZ). The governance structure consisted of a Project Lead, a Project Team (including two sub-teams, one on process and one on methodology), a Steering Committee and an Advisory Group. In addition, ZIN has designated in total 11 staff members for the HTACG and its subgroups and the HTA Comitology Committee (the HTA Comitology Committee is a committee supporting the European Commission with the development of the Implementing Regulations. All EU MSs and Norway are represented in this committee).

Table 1 describes in more detail how many individuals from which departments were involved in each part of the governance and what their key tasks were.

### 3.4. Exchange with External National Stakeholders

Part of the activities was to exchange with external national stakeholders in various formats, as appropriate. ZIN has engaged in exchanges the association of innovative medicines developers, the healthcare insurers, patient organisations and clinical societies for the new situation and the role they were anticipated to have or how it will impact them. Table 2 presents the various types of meetings ZIN had with the different external stakeholders.

### 3.5. Potential Gaps, Challenges and Issues

All topics identified in the gap analyses were classified into the following categories: legal, information and knowledge, IT and template, communication and stakeholder engagement, capacity and resources, and financial aspects. Section 3.5 explains the gaps, challenges or issues we have identified, and in Section 3.6 the actions taken or suggested to address the gaps, challenges or issues are presented.

#### 3.5.1. Legal

An important finding was that no changes to Dutch legislation were required to participate in the HTAR activities, mainly due to two reasons: (1) European regulations automatically apply to EU MS without the need for transposition into national law (Article 288 of the Treaty on the Functioning of the European Union [13,14], and (2) the Dutch HTA process is governed only in general terms under the Health Insurance Act. This act does not impose specific requirements (e.g., on the source of evidence for clinical effectiveness) that would hinder ZIN’s contribution to the HTAR or give due consideration to the JCA.

#### 3.5.2. Information and Knowledge

To determine whether ZIN will take on the role of (co-)assessor of a specific JCA, an additional step must be added to its current national agenda-setting process. Given ZIN’s limited capacity to take on (co-)assessor roles for JCAs, the challenge for ZIN is to gauge, at an early stage, whether the medicinal product will require mandatory assessment by ZIN. In the current situation, ZIN is informed about this by their own horizon scanning activities. In the new situation, the JCA will start in parallel with the regulatory process. Therefore, the decision on whether a product will likely be within the scope of ZIN has to be made much earlier and alignment with the national horizon scanning activities is crucial.

Before the national assessment process can start, the dossier submission of the HTD to ZIN needs to be declared “complete”. A dossier can only be declared complete once the JCA report has been uploaded to the IT platform, as it must be part of the national report.

An important change is that ZIN can only request additional information from the HTD that has not yet been shared at EU level. Examples of additional information required for the national decision-making process include a pharmaco-economic dossier and budget impact analysis. Using the JCAs in practice, we will learn how often additional information is needed for the national process and how the HTAR process will impact this. In the Netherlands, the HTD has the opportunity to submit a preliminary dossier for the national assessment process. The exact timing of this submission greatly depends on when the HTD decides to submit to ZIN, but the preliminary dossier procedure allows for early submission. The definition of ‘early’ needed to be revisited, considering the timelines of the JCA process.

The Netherlands is member of the Beneluxa Initiative, a collaboration with Belgium, Luxemburg, Austria and Ireland. Within the national legal frameworks, a joint Beneluxa assessment can be performed using the submission of the same file (in English) in all participating countries as the starting point [15]. The availability of JCAs might impact the way of working of Beneluxa.

Finally, all staff involved in conducting JCAs should be well trained in the methods included in the adopted HTACG methodological guidance documents and the full JCA procedure. Knowledge gaps were specially identified in the areas of indirect treatment comparisons and network meta-analysis.

#### 3.5.3. IT and Template

All HTA bodies in the EU MS need to give “due consideration” to the JCA report. Due to the clinical and objective remit of the JCA, value judgements on the overall clinical value of the assessed product will have to be made in the national assessment report. It is expected that the template of the national pharmacotherapeutic dossier will largely be suitable for incorporating the evidence from the JCA report, with a few minor adjustments to accommodate additional evidence reported in the JCA report. In addition, a decision needs to be made about the language in which the national reports will be written to make sure these reports meet the national preferences and requirements and safeguards the efficiency of the process.

Related to the HTAR’s IT platform, two major issues were identified. First, to be able to access and use the documents, all those involved in the JCA need to have access to the IT platform. Second, it should be crystal clear which information from the national HTA report ZIN has to upload on the IT platform. See Annex I for the MS obligations on uploading information on the IT platform as outlined in the HTAR (see Appendix A).

#### 3.5.4. Communication and Stakeholder Engagement

The period for ZIN to respond to the PICO proposal made by the JCA assessor and co-assessor is relatively short. As ZIN uses input of physicians’ and patient’s associations in the national scoping [16], these organisations need to be contacted promptly, and a procedure is needed for quickly collecting their input. ZIN needs to manage expectations, because stakeholders may feel the obligation or need to have an opinion of how the new product should be positioned in the treatment landscape, whereas ZIN primarily needs to collect information on relevant comparators.

Deciding on the communication language of the national process is an issue that affects the work of ZIN’s employees. Currently, the national reports are written in Dutch, and only ZIN’s advisory letters are translated into English and published on ZIN’s website. Striking the right balance between efficiency gains from using the English language and transparency towards Dutch citizens and patients is seen as a challenge.

ZIN also identified an issue related to the communication between ZIN and the Dutch health insurers in case a JCA of a medicinal product falls outside ZIN’s assessment scope. Then it will be relevant to health insurance companies who are responsible for the reimbursement decision in that situation. It is unclear what role ZIN and the health insurance companies using HTA information should assume in those situations. If ZIN is going to be the intermediate between the EU HTA process and the health insurers, a new process and line of communication needs to be developed considering the confidentiality and conflict of interest rules under the HTAR.

#### 3.5.5. Capacity and Resources

Being structurally involved in the JCA at the EU level represents an addition to the current national HTA process that requires additional capacity. In the long term, however, the burden on capacity is expected to decrease as more JCA reports (written by other countries) can be used on the national level. Following the criteria defined in the HTACG guidance on the appointment of the assessor and co-assessor for JCA, the assessor or co-assessor needs to include an information specialist and statistician. In the Netherlands, this requires an increase in national capacity for this type of expertise. Especially, statistical expertise and expertise in indirect treatment comparisons and network meta-analysis need to be strengthened as an increase in this type of evidence is expected to meet the anticipated large number of PICO requests from the Member States.

Furthermore, additional human resources are required to ensure that the JCA reports are given due consideration in the national process, and that the national PICO(s) for the JCA and the national assessment reports are prepared.

#### 3.5.6. Financial Aspects

The funding for additional staff must align with the ambitious role ZIN aims to play in the HTAR. At the same time, the ambitions must be adjusted to match the available resources given the current national workload. Clarity remains to be provided by the sustainable financing model that is anticipated under the HTAR Article 27 [1].

### 3.6. Actions and Adjustments to the National Process

Table 3 summarises the actions that were taken to address the gaps described above. It should be noted that some of the anticipated gaps and challenges were resolved over time as the details of the EU HTA process became clearer and more clarity emerged about ZIN’s own ambitions.

## 4. Discussion

In this paper, we have described the results of the preparatory activities of ZIN to implement the HTAR in the Dutch setting by applying structured change management to achieve the desired outcome of a national HTA process that enables efficient contributions to the JCA process and efficient use of the JCA report in the national HTA process. A key activity to achieve this was the conduct of a practical gap analysis. Part of the success of our preparatory activities was the great number of meetings with external stakeholders to raise awareness about the changes coming up and to facilitate a dialogue on appropriate actions to be taken on the identified gaps, challenges or issues.

Topics that arose from a gap analysis were grouped into six domains, i.e., legal, information and knowledge, IT and template, communication and stakeholder engagement, capacity and resources, and financial aspects. We have described the actions taken to address the potential gaps, issues or challenges, including adaptations to the national HTA process. A horizontal project structure was created to govern the preparatory work and involve all relevant individuals from different departments at ZIN. This governance structure worked well, although it is advised to consider a somewhat leaner structure for future implementation of new regulations. The current structure was sometimes leading to duplication of people involved in different groups, and it also was a long and intense process. Nevertheless, it is a very valuable structure to ensure that information was being shared with relevant colleagues within the HTA body and this positively influenced the acceptance of adaptations to the national HTA process. In addition, ZIN has benefitted greatly from its active role in EUnetHTA, particularly in EUnetHTA 21, which gave implementation of the HTAR at the national level a head start. ZIN’s early involvement in the HTACG, its subgroups and the HTA Comitology Committee also offered continuous insights and important updates throughout the development of the methodological guidance and the implementing regulation. All the preparatory work has led to ZIN feeling well prepared to take on (co-)assessor roles for the JCA of medicinal products.

This entire process of thorough preparation shows a strong level of support for the goals of the HTAR and confidence in its success. This ambition is driven by the conviction that the HTAR will enhance the efficient use of HTA resources and improve the quality of HTA across the EU, with the aim of reducing variations in reimbursement decisions and, consequently, the availability of interventions across MSs.

ZIN started the gap analysis rather early, before all the details on the process and methodology were known. Therefore, the gap analysis followed an iterative process and some of the gaps initially identified were closed as the implementation of the HTAR became clearer. While the action list in the article shows that many issues have been adequately addressed, there also remain a few outstanding topics that require further action or clarity.

### 4.1. Applicabitlity for Other MSs

Although there is great divergence in HTA organisations across Europe in their scope and remit and also in the process of national HTA, there are similarities as well, for example, in the starting point and data used for the national HTA [8,19,20]. Given that all the EU and EAA MSs have to comply with the HTAR and that the scope of the HTAR will expand over the coming years, the process, governance and identified gaps described in our paper seem relevant for other EU HTA bodies as well. The extent to which the individual gaps and actions described may be applicable to other MSs depends on the details of their respective HTA system; however, the main themes we identified in our paper will be of relevance. Regardless of the identified gaps, challenges and issues, the methods and process that ZIN used to manage the changes are less dependent on the actual healthcare system.

### 4.2. Continuous Monitoring and Evaluation

The success of the HTAR depends on the cooperation of all MSs, which must adapt their national assessment and reimbursement processes [6,21,22] and are likely to learn from best practices in other MSs [23]. The success also depends on the collaboration with the stakeholders involved, recognising that their capacity to contribute is constraint as well [22]. Efficient processes are expected to emerge along the way, building on the experiences in the coming years. An evaluation of the HTAR is foreseen and should be ready in 2028 [1]. This makes it important for MSs to closely monitor the implementation process and outcomes.

From its early involvement in the EUnetHTA Joint Actions [2,3,4], ZIN believes that HTA collaboration at the European level will speed up access to real innovation, harmonize methods for clinical aspects of the HTA and reduce duplication of work. However, there is also a clear understanding that achieving these goals will take time. ZIN’s expectation is that, whilst the HTA process will initially take more time, efficiency gains will be realised as more experience is gained in defining PICO’s and integrating the JCA into the national approval process. It thinks that the timely finalisation of the JCA, namely 30 days after Marketing Authorisation, in conjunction with the adapted national process, will aid the national uptake of the JCA reports, assuming the HTD will follow with a timely national submission. However, ZIN will continue to monitor the national implementation of the HTAR to assess whether the aims are achieved.

Next to this, whether the process for consulting national patient organisations and clinical societies is feasible in the very short timeline that is available must be monitored. Fortunately, in the Netherlands, all organisations are on the radar, as they have an active role in the Dutch process. They are involved in the scoping phase and are asked for written consultations on the draft national HTA reports and for live consultations during the ACP meetings. This ensures shorter communication lines compared to countries without an established process for stakeholder consultation.

For the time being, it was decided to continue to write national HTA reports in Dutch. However, in the future this could be re-considered. Writing in English would make the national assessment process more efficient, as sections from the JCA could be easily copied and pasted into the pharmacotherapeutic report. Furthermore, it would facilitate the exchange of information from reports between countries. On the other hand, Dutch citizens might find it harder to understand the national reports. Finally, engaging patient organisations in the national HTA process may be impacted as it may be more difficult for them to understand the original documents.

A continuous point of attention is that the current Dutch process might be subject to change, which might impact the implementation process of the HTAR as well. For example, at the moment, the process for an HTD to start the preliminary dossier procedure is being adjusted. The impact of such changes on the HTAR implementation must be taken into account.

While the JCA only describes the clinical effects without value judgements, the PICOs requested by MSs may be based on economic arguments. Not all HTDs might be sufficiently aware of this and it may give rise to discussions that need to be closely monitored. For example, a cheaper and perhaps off-label comparator can be part of the standard care or certain outcome measures can be particularly relevant for the pharmacoeconomic assessment. As per scoping guidance adopted by the HTACG, off-label comparators can be considered in the PICO(s) for the JCA [24].

### 4.3. Future Expansion

While the HTAR and the JCA Implementing Regulation of medicinal products foresee the possibility for re-assessments or updates of JCAs, ZIN has not addressed this in its implementation project. The reason is that no updates to JCAs are expected during the initial period of the HTAR [25]. For such updates, it is expected that part of the evidence will be real-world evidence (RWE). In the published guidance on the validity of clinical studies for JCAs adopted by the HTACG, it is described—only at a high level—how RWE could be included in a submission dossier and how it could be reported [26]. Additionally, the HTACG adopted guidance on how to fill the submission dossier template and includes flow charts on when RWE could be presented [27]. The role of RWE is likely to become more important as RWE is expected to increasingly fill in the gaps for PICOs with little or no evidence from randomised controlled studies. ZIN has a history of experience with re-assessments based on RWE in the context of conditional reimbursement agreements for expensive and orphan drugs. However, since the introduction of the lock procedure, in 2015 the number of these agreements has been very limited [28]. Re-assessments in the context of European JCAs is a typical example of a topic that is expected to evolve over time.

While ZIN’s national implementation project mainly focused on getting ready to conduct and use JCAs for medicinal products, aspects around medical devices were also discussed. Nevertheless, ZIN should conduct a similar exercise for JCAs of MDs and IVDs as the templates and timelines are expected to be different on both a European and national level. This is scheduled for 2025 as the first JCAs of MDs and IVDs are expected to start in 2026. However, the number of such JCAs is expected to be low as only those MDs that will receive an expert panel opinion or IVDs that will receive an expert panel view of the clinical evaluation consultation procedure will be eligible for JCAs (HTAR, Article 7 [1]).

Additionally, ZIN should further investigate their ambition and possibilities to engage in Joint Scientific Consultation (JSC) activities (additional to the participation in the JSC subgroup). In the JSC phase of the HTAR, the HTD can request scientific advice on the clinical study plan. ZIN acknowledges the importance of the JSC for adapting the study designs to HTA bodies’ needs [29], but due to capacity constraints it decided to concentrate on the JCA now. For ZIN to contribute to the JSC as (co-)assessor, staff with expertise in clinical study design and outcome measurement is required. This expertise might not be sufficiently present because the European JSC is more extensive than the current Dutch early dialogue, which mostly focusses on dossier-related aspects rather than fundamental questions on study design. Furthermore, in the Netherlands, the early dialogue is optional, and relatively few such advisory meetings are held. Hence, the number of assessors with the required expertise might need to be increased, depending on the time required by each JSC and the ambitions of ZIN’s involvement in the both the JCAs and JSCs.

## 5. Conclusions

Due to ZIN’s substantial investment in preparations for the HTAR, it is prepared for and ready to implement the HTAR in the national HTA processes and to take part in the HTACG and its subgroups. Furthermore, ZIN is prepared to take on assessor or co-assessor roles for JCAs of medicinal products from 2025 on. For this reason, two assessors were hired for the purpose of European HTA work. It remains crucial to monitor the revised national process for feasibility and efficiency, even though a more efficient process is expected under the HTAR in the long term. The governance structure used for the HTAR national implementation project was useful, but it is recommended to adopt a leaner structure for any further implementation of new regulations. In light of the future expansion of the EU HTAR, it is important to continue sharing these implementation experiences between MSs.

## Figures and Tables

**Table 1:** Governance of the preparations for the implementation of the HTAR at ZIN.

	Participants Involved	How Often Did They Meet	Key Tasks
Project Lead	1 project lead, the coordinator of ZIN’s international affairs	N/A	Leading the entire project; ensuring timelines are met and everyone is well informed
Project Team	10 individuals, including 4 individuals experienced in performing HTA of medicinal products, and 6 individuals representing broader HTA and methodological and procedural knowledge and experience, also in the field of MD/IVD	Every month	Monitoring the process and upcoming activities, working in sub-teams
Two project sub-teams, one on process and one on methodology	Both sub-teams consisted of 3–4 individuals from the Project Team or additional staff members from the department performing the HTA	Bi-weekly	Conducting the respective gap analysis and adapting the national procedures and methodologies as required; depending on the questions at hand, colleagues from other relevant departments collaborated, e.g., IT, legal and communication colleagues
Steering Committee	3 individuals, on a senior management level, including directors, from relevant departments	Every 2–3 months, as needed	Monitoring the progress and deciding on key questions/steps forward, also based on discussions that took place in the HTA Coordination Group or its subgroups
Advisory Group	Around 15 individuals (some overlap with the project team); this group included individuals from, for example, the medicines HTA team, the legal team, communications department and IT services	Every 6 weeks	Sharing information, collecting input and gaining broad support within ZIN
Internal meetings with the ZIN representatives in the HTACG, the four subgroups and the HTA Comitology Committee	In total, 11 experienced individuals participate in these groups in a fixed allocation as per the HTACG Rules of Procedures	The HTACG meets 4–5 times per year, the subgroups meet every (other) month and the HTA Comitology Committee meets every month if needed; the ZIN organises once every month a preparatory and a debriefing meeting	The preparatory and debriefing meetings are meant to inform each other and to align the views and agree on decisions to be made (e.g., adoption of guidances) and share feedback on how to further improve the process; this was also used to align on feedback for the guidances prepared by the subgroups, to ensure all relevant information necessary for ZIN would be obtained

**Table 2:** Exchange with external stakeholders.

Stakeholder Group	Attendees and Purpose of Meeting
National association of innovative medicine developers	The HTA taskforce of the Dutch Industry Association of Innovative Medicines (consisted of around 6 individuals) and the sub-project team on process and methods. Regular meetings to continue the discussions on relevant questions for the national HTA procedure, such as timelines and the national submission process. Additionally, in Q2 2024 a workshop was organised with over 50 representatives from national pharmaceutical industry organisations, as well as representatives from national industry associations. The purpose of the workshop was to inform all relevant Dutch industry organisations about the anticipated changes on the Dutch level, for example, the scoping and procedures as well as the anticipated national timelines for submitting a national dossier on a product that was subject to a JCA.
Patient organisations	Regular meetings with representatives from patient organisations to inform them about ZIN’s preparatory activities. In Q4 2024, a workshop was organised for patient organisations in the field of oncology and ATMP to inform them about the envisioned national process for engaging patient organisations when a JCA is taking place.
Clinical societies	ZINs preparatory activities around the HTAR implementation, specifically around the engagement of clinical societies during a JCA were discussed during national virtual meetings.
National healthcare insurers	Regular meetings with the Committee Assessment of Add-on Medication (CieBAG) on the anticipated changes due to the HTAR, and their representatives also have joined workshop hosted by ZIN, for example, on the scoping process. Section 3.1 further explains the role of the CieBAG in the Netherlands.
HTA info days	This info day was organised by the European Commission, together with the Beneluxa countries, and it was part of a series of in total six HTA info days. The purpose was to raise awareness on the HTAR and the anticipated changes on the national level. On behalf of the Netherlands, 30 people were invited to join the meeting in person. The meeting was also live broadcasted and the recording is still available (https://health.ec.europa.eu/events/theory-practice-implementing-eu-health-technology-assessment-regulation-2024-01-30_en). The Dutch attendees were 8 clinicians, 7 governmental representatives (including ZIN, Ministry of Health and the Dutch Medicines Evaluation Board CBG), 4 health insurers, 5 industry HTD representatives from umbrella organisations for medicinal products and medical devices and 6 patient representatives.

**Table 3:** Action plan to address the gaps, challenges and issues.

Type of Gap, Challenge or Issue	Action Taken
Legal	No actions required at the start of the project; however, ZIN should further investigate the potential impact of the Implementing Regulations, specifically the one on Conflict of Interest [17].
Information and knowledge	Brainstorm with the Horizon Scanning team on how to obtain information in a timely manner to decide on ZIN’s involvement in JCAs and create an internal working agenda. It was decided that an HTD can start the preliminary dossier procedure once the submission dossier at EU level is declared complete and before the JCA report is finalised. This timing has been chosen to avoid any duplication of efforts for the HTD while they are working on the EU submission dossier, but, ultimately, it is up to the HTD to request this process and timely inform ZIN about plans to do so. Clarify the type of additional information beyond the JCA that ZIN will need to ask the HTD to include in their submission. Discuss the HTAR within the Beneluxa domain task force on HTA. It is expected that a European JCA might facilitate the joint work as it provides a good starting point. Provide training in advanced statistics, indirect comparison/network meta-analysis and ROBINS-I. Investigate if other training opportunities, e.g., via SUSTAIN-HTA and HAG-INSIGHT, can meet the training needs in areas ZIN is less experienced in. Investigate if training in medical writing in English is needed; in the end, it was not necessary. Participate in a European workshop on scoping and participate in the PICO exercises conducted in the JCA subgroup. Conduct national PICO workshops. Develop a process to quickly gather input from relevant stakeholder organisations because the time to respond to PICO requests is short. Set up workshops to develop a process for implementing information from the JCA report into the national HTA report and to practice with the development of PICOs.
IT and template	Complement the handbook on assessment with the additional steps resulting from the HTAR. Adapt the checklist of dossier completeness required to start the national HTA process. Redesign the templates for dossier submission to align with the JCA report and explain how the JCA was given ‘due consideration’. Check the compliance of the internal IT structure with the EU IT platform.
Communication and stakeholder engagement	Engage with patient and professional organisations to inform them about the upcoming legislation and discuss how they can be involved in the process of PICO-defining. Decide on the language of the national HTA reports. It was decided that the national report will remain in Dutch for now. Meetings with CieBAG (health insurers) on how ZIN could relate to their need to use the JCA report in their HTA process. Still, more clarity is required on appropriate confidentiality measures in case the health insurers should be involved during the JCA report. Meet with the national HTD umbrella organisations to discuss the timing of sharing the PICO in their reimbursement application as well as timing of the submission of the national dossier. It was decided to share the European PICO requested by the Netherlands after the HTD has submitted the JCA dossier. Contribute to national and international symposia, conferences, seminars to raise awareness of the HTAR.
Capacity and resources	Write an ambition memo for ZIN’s Steering Committee and Board of Directors. ZIN has decided to aim for taking on the role of (co-)assessor in the JCAs of medicinal products a few times. Use timesheets from ZIN based on EUnetHTA participation to estimate the mean time required to fulfil each role in the JCA. This information is used to clarify ZIN’s ambition regarding involvement in JCAs as assessor or co-assessor. Secure availability of inhouse statistical expertise and train ZIN’s information specialist to participate in JCAs. Recruit two additional assessors to enhance capacity for contributing to the writing of the JCAs. Assessors involved in the JCA should be flexible, creative and stress-resistant as experience with the new process needs to be gained. These assessors are hired specifically for the JCA work but may be involved in the Dutch HTA process in less busy times of the JCA work
Financial aspects	Explore the possibilities to financially support the additional capacity needed to facilitate ZIN’s ambitions for involvement in JCAs. Await results of the awarded European Commissions’ tender for financial reimbursement for the roles of assessor and co-assessor [18]

## Data Availability

The original contributions presented in this study are included in the article. Further inquiries can be directed to the corresponding author.

## Abbreviations

The following abbreviations are used in this manuscript:

ACP	Insured Package Advisory Committee
ATMP	Advanced Therapeutic Medicinal Products
CD	Commission Decision on Marketing Authorisation
CHMP	Committee for Human use of Medicinal Products
CieBAG	Committee Assessment of Add-on Medication
EMA	European Medicines Agency
EU	European Union
EUnetHTA	European Network for HTA
HTA	Health Technology Assessment
HTACG	HTA Coordination Group
HTAR	HTA Regulation (EU) 2021/2282
HTD	Health Technology Developer
IVD	In-Vitro Diagnostic MD
JCA	Joint Clinical Assessment
JSC	Joint Scientific Consultation
MAA	Marketing Authorisation Application
MD	Medical Device
MS	Member State
OWIZ	Development & International Affairs
PICO	Patient/Intervention/Comparator(s)/Outcome(s)
WAR	Scientific Advisory Board
ZIN	Zorginstituut Nederland; the Dutch National Healthcare Institute

## Appendix A

**Table A1:** MS obligations according to the Regulation (EU) 2021/2282 Article 13 [1].

Article	Text	Explanation
Article 13.1(a)	Member States shall give due consideration to the published JCA report and all other information available on the IT platform (…)” but it “shall not affect Member States’ competence to draw their conclusions on the overall clinical added value of a health technology in the context of their specific healthcare system (…).”	This ensures that a MS has to use the JCA, while at the same time the national authority is not restricted. As it is mandatory for MS to give due consideration to the JCA report, they are responsible for incorporating the European report in their national HTA procedures.
Article 13.1(b)	“annex the dossier submitted by the health technology developer (…) to the documentation of the HTA at Member State level”	This requires that the EU JCA submission dossiers has to be annexed to the national HTA documentation. It is anticipated to increase transparency of the evidence and information available to the JCA and is in the interest of public health.
Article 13.1(c)	“annex the published joint clinical assessment report to the HTA report at Member State level”	The addition of this annex is anticipated to increase transparency on how the JCA report has been used, and it enables active references to the JCA report without unnecessary copy/pasting of information.
Article 13.1(d)	“not request at the national level information, data, analyses or other evidence that has been submitted by the health technology developer at Union level (…).”	This ensures that the health technology developer does not have to submit the same information twice.
Article 13.1(e)	“immediately share through the IT platform (…) any information, data, analyses and other evidence with the Coordination Group that they receive from the health technology developer at Member State level and which form part of the submission request made pursuant to Article 10(1).”	This obligates MSs to share relevant evidence and information which was requested during the JCA but not received.
Article 13(2)	“Member States shall provide the Coordination Group through the IT platform (…) with information on the national HTA in respect of a health technology which has been subject to a joint clinical assessment within 30 days from the date of its completion. (…)”	This obligates MSs to provide documentation on results of the national HTA process (i.e., the HTA advice).
